# Modulation of digestibility of canine food using enzyme supplement: an *in vitro* simulated semi-dynamic digestion study

**DOI:** 10.3389/fvets.2023.1220198

**Published:** 2023-08-09

**Authors:** Swati Jadhav, Tejal Gaonkar, Mithila Joshi, Abhijit Rathi

**Affiliations:** Food Application and Development Laboratory, Advanced Enzymes Technologies Ltd., Thane, India

**Keywords:** canine food, enzyme blend, macronutrient digestion, semi dynamic *in vitro* digestion, antioxidant

## Abstract

Digestibility and nutrient availability are important parameters when estimating the nutritional quality of pet food. We have developed a simulated semi-dynamic *in vitro* canine digestion model to evaluate the digestibility of dry extruded canine food. Canine food was assessed for digestible energy, dry matter digestibility, protein digestibility, non-fibrous carbohydrate (NFC) digestibility, and total antioxidant capacity (TAC) in the absence and presence of an enzyme blend (DigeSEB Super Pet). Enzyme blend supplementation in canine food was found to increase the dry matter digestibility (18.7%, *p <* 0.05), digestible energy (18.1%, *p* < 0.05), and protein digestibility (11%, *p <* 0.1) and reducing sugar release (106.3%, *p <* 0.005). The release of low molecular weight peptides (48.7%) and essential amino acids (15.6%) increased within 0.5 h of gastrointestinal digestion due to enzyme blend supplementation. Furthermore, the TAC of the digesta was also increased (8.1%, *p* < 0.005) in the canine food supplemented with enzyme blend. Overall, supplementation of enzyme blend in canine food is an effective strategy to enhance the food digestibility and nutrient availability for absorption.

## 1. Introduction

Companion animals positively affect the emotional and physical health of people with whom they are in contact. Anthropomorphism of canines makes owners more concerned about their pet’s health and wellbeing (1). They are observant of their pets’ diet in order to provide optimal nutrition and maintain their long-term health (2). Digestibility and nutrient availability are important parameters when estimating the nutritional quality of pet food (3). The diet composition, nutrient availability, and their interaction also regulates the cognition and behavior of canines (4, 5).

The pet food industry has introduced various commercial extruded kibble diets to the market (Pedigree adult chicken and vegetable, Ykibble oven baked premium canine food, IMS proactive health, etc.) with beneficial claims (improved skin health, strong bones and teeth, strong muscles, natural defense, optimum health, etc.). Health and nutrition are the foremost criteria of pet food selection; there are also quality, ingredients, freshness, taste, pet preference, and ease of preparation (6). A variety of plant-based (fruits, vegetables, grains, legumes, nuts, and seeds) and animal-based (meat, eggs, dairy products, and organ meat) ingredients are added to the pet food (7) either individually or in combination to provide a ‘complete and balanced’ diet that meets the nutritional requirements. However, adequate digestion of the macromolecules (carbohydrate, protein, and fat) in the diet is imperative to disintegrate the food matrix and to release the required macronutrients, micronutrients, and minerals from the diet for absorption. While manufacturing commercial canine food products, processing methods positively or negatively affect the nutritional value (8). For instance, extrusion cooking positively influences palatability, digestibility, and destruction of undesirable factors but can also have a potentially negative impact on protein quality and vitamin availability (9). The inclusion of vegetable-based ingredients may add some anti-nutritional factors to the canine diet (10). Additionally, digestive health varies in each canine along with factors such as age and other illnesses. In this scenario, pet food supplemented with enzymes can enhance its digestibility and nutrient availability. A multitude of enzymes (β-mannanase, phytase, protease, xylanase, β-glucanase, cellulase, amylase, pectinase, lipase, and glucoamylase) have been studied to evaluate their effect on the digestibility of canine food (11–18). The inclusion of mannanase in the soybean meal increased protein and energy digestibility in dogs (11). The addition of proteases and lipases in feather meal showed enhanced digestible energy in dog trials (15). Diets supplemented with xylanase, β-glucanase, and amylase alleviated the anti-nutritive effect of non-starch polysaccharides (16). Further, multi-enzyme complexes are known to improve nutrient digestibility in pigs and poultry (19–23).

Here, we developed a simple and reproducible simulated semi-dynamic *in vitro* canine digestion model to study the canine food digestibility in presence of an enzyme blend (EB) supplement. We hypothesized that canine food supplemented with EB would enhance the digestibility and release of nutrients compared to its non-supplemented counterpart. DigeSEB Super Pet (a commercial enzyme blend) was used in this study as a model enzyme blend supplement.

## 2. Materials and methods

### 2.1. Materials

Extruded dry adult canine food [protein 21.5%, fat 7.4%, carbohydrate (nitrogen-free extract + crude fibers) 55.9%, moisture 8.9%, ash 6.3%, and gross energy 4.4 kcal/g; Ingredients: cereal and cereal by-product, chicken and chicken by-product, meat and meat by-product, soybean meal, di-calcium phosphate, soyabean oil, iodized salt, choline chloride, vitamins and minerals, antioxidant, carrot powder, pea powder, zinc sulfate monohydrate, preservative, and flavors] was purchased from the local market. Enzyme blend (DigeSEB Super Pet: acid proteases 10,500 HUT/g, alkaline proteases 1800 PC/g, amylase 2,135 SKB/g, and lipase 155 FIP/g) was a gift sample from Specialty Enzymes, United States. Pepsin (P6887) and pancreatin (P7545) were purchased from Sigma, India. Other chemicals used were of AR grade and purchased from Merck, India.

### 2.2. Simulated semi dynamic *in vitro* digestion model

A simulated semi dynamic canine *in vitro* digestion model was developed using the information obtained from the dynamic digestion model described by Smeets-Peeters et al. (3). The gastrointestinal digestion of the canine food was performed in a 2 L glass reactor with a temperature set to 39°C and a pH probe inserted in the reactor for pH monitoring. Briefly, food solution (150 mg of finely ground food powder/mL of distilled water, 300 mL) was mixed with simulated gastric fluid, pH 1.9 (10 mL; SGF-NaCl 3.5 g/L, KCl 1.3 g/L, CaCl_2_ 0.2 g/L, NaHCO_3_ 0.25 g/L, pepsin 75 mg/L, and lipase 90 mg/L), and EB (1% of the food). The control reaction was set up by replacing EB with an equal amount of distilled water. Gastric digestion was carried out at 39°C and 100 rpm for 3 h. Kinetic aspects of the dynamic model, such as gradual acidification and fluid and enzyme secretion, followed in the gastric phase of this model. After every 0.5 h of the gastric phase 15 mL of SGF pH 1.9 was added to the reaction mixture, and the pH of the system was adjusted using 1 N HCl. The pH was adjusted to 5.4, 5, 4.2, 3, 2.3, 2.1, and 1.9 at 0, 0.5, 1, 1.5, 2, 2.5, and 3, respectively (Supplementary Figure 1). Samples (20 mL) were removed at 0.5, 1, 2, and 3 h of the gastric phase, and the reaction mixture was replenished with the same amount of SGF. After 3 h of gastric digestion, the entire gastric digesta was shifted to another 2 L glass reactor (set at intestinal reaction conditions). Gastric digesta was mixed with the 417 mL of the simulated intestinal fluid (Supplementary Figure 1) (SIF-NaCl 7 g/L, KCl 0.5 g/L, and MgCl_2_.6H_2_O 0.813 g/L), 135 mL of bile solution (60 g/L), and 67 mL of pancreatin (10 g/L). The pH of the reaction was adjusted to 6.5 using 1 N NaOH. The intestinal digestion was further carried out at 39°C and 100 rpm for 3 h. Samples (20 mL) were removed at 0.5, 1, 2, and 3 h of the intestinal phase (Gastrointestinal or GI phase), and the reaction mixture was replenished with the same amount of SIF. All the samples were centrifuged at 4°C and 3,000 rpm for 10 min. Pellet and supernatant were separated and used as undigested and digested fractions, respectively. Obtained undigested fractions were dried at 65°C until they reached a constant weight (24). All the digested fractions were stored at −20°C until required for analysis.

### 2.3. Analysis

#### 2.3.1. Dry matter digestibility and energy digestibility

Moisture content of the undigested fraction was determined using IR balance, and dry matter digestibility was calculated using the following formula (24).

$$\begin{array}{l} Dry\;matterdigestibility\;\left(\%\right)=\\ \frac{\mathrm{Weight of}\;\mathrm{raw}\;\mathrm{sample}-\mathrm{Weight of undigested fraction}}{Weightof\;raw\;sample}*100 \end{array}$$

The energy content of the raw sample and undigested fraction was determined using an automatic Hamco 6E bomb calorimeter (25). Digestible energy was calculated using the following formula.

$$\begin{array}{l} Digestibleenergy\;\left(kcal/kg\right)=\\ Grossenergyof\;raw\;sample-\\ Grossenergyofundigestedfraction \end{array}$$

#### 2.3.2. Protein digestibility

EB blanks were run for each test but the protein was too low to contribute in any of the test results. The total protein present in the raw sample and undigested fraction was determined using the Kjeldhal method (6.25 conversion factor), and the protein digestibility was determined using the formula below (26, 27):

$$\begin{array}{l} Proteindigestibility\;(\%)=\\ \frac{\begin{array}{l} (Totalproteinin\;raw\;sample\;(g)-\\ Totalproteininundigestedfraction\;(g)) \end{array}}{Totalproteinin\;raw\;sample\;(g)}*100 \end{array}$$

The digested fraction (digesta) was analyzed for the degree of hydrolysis using an o-phthalaldehyde (OPA) assay. The sample (25 μL) was mixed with the OPA reagent (175 μL), and the reaction was incubated at room temperature (27 ± 2°C) for exactly 2 min. Absorbance was measured at 340 nm (28). Free amino groups were determined using slope of the standard curve (40–200 μg/mL of serine). The raw sample was hydrolyzed by acid and evaluated for total free amino groups by OPA assay. The degree of hydrolysis was determined as follows:

$$\begin{array}{l} Degreeofhydrolysis\;\left(\%\right)=\\ \frac{Freeaminogroupsinthedigesta}{Freeaminogroupsinacidhydrolysed\;raw\;sample}*100 \end{array}$$

Amino acids released in the digesta were determined using HPLC with DAD detector. The column used was Agilent Zorbax Eclipse AAA at 40°C with a flow rate of 2 mL/min. The samples were derivatized using o-phthalaldehyde (OPA) and fluorenylmethyloxycarbonyl chloride (FMOC) as per the Agilent’s instruction manual. The gradient system started from 98% of 40 mM phosphate buffer pH 7.8 and ended with 2% of an acetonitrile:methanol:water (45:45:10) mixture. Molecular weight distribution of the peptides in the digesta was determined using a SEC-HPLC system (28). Appropriately diluted samples were run on BioSep, 5 μm, SEC-s2000, 145 Ǻ (Phenomenex Inc.) column at 25°C with a flow rate of 1 mL/min. The mobile phase used was a phosphate buffer (0.1 M, pH 6.8), and peptides were detected using DAD at 214 nm.

#### 2.3.3. Non-fibrous carbohydrate digestibility

The total reducing sugars released in the digesta were quantified using 3,5-Dinitrosalicylic acid (DNSA) method (29) and dextrose (0.1–1 mg/mL) as a standard. Glucose released in the digested samples was detected using a GOD-POD kit (AUTOSPAN® liquid gold glucose kit).

#### 2.3.4. Total antioxidant capacity

The total antioxidant capacity (TAC) of the digesta was determined using a phosphomolybdate assay and ascorbic acid (40–200 μg/mL) as a standard (30). Briefly, an appropriately diluted sample (0.1 mL) was mixed with the phosphomolybdate reagent (1 mL, 4 mM ammonium molybdate and 28 mM sodium dihydrogen phosphate in 0.6 M sulfuric acid). Reaction was incubated at 95 ± 2°C for 90 min, and absorbance was noted at 765 nm. TAC of the digesta was calculated as ascorbic acid equivalent in total digesta (mg) using following formula:

$$\begin{array}{l} \mathrm{Ascorbic acid equivalent in total digesta}\;\left(\mathrm{mg}\right)=\\ \left(\frac{\left(T-B\right)-constant}{Slopeofstandardcurve}*D\right)*\frac{V\;}{1000\;} \end{array}$$

where T and B-absorbance of test and reagent blank at 765 nm, D—dilution factor, and V—volume of the digested sample (mL).

#### 2.3.5. Statistical analysis

All the experiments were performed in triplicates and represented as mean ± SD. Statistical analysis was performed on GraphPad Prism 9. Student’s *t*-test and two-way ANOVA with Tukey’s multiple-comparison test were used to analyze the data. *p* ≤ 0.05 was considered as statistically significant.

## 3. Results and discussion

### 3.1. Simulated semi dynamic *in vitro* digestion model

**Figure 1:**
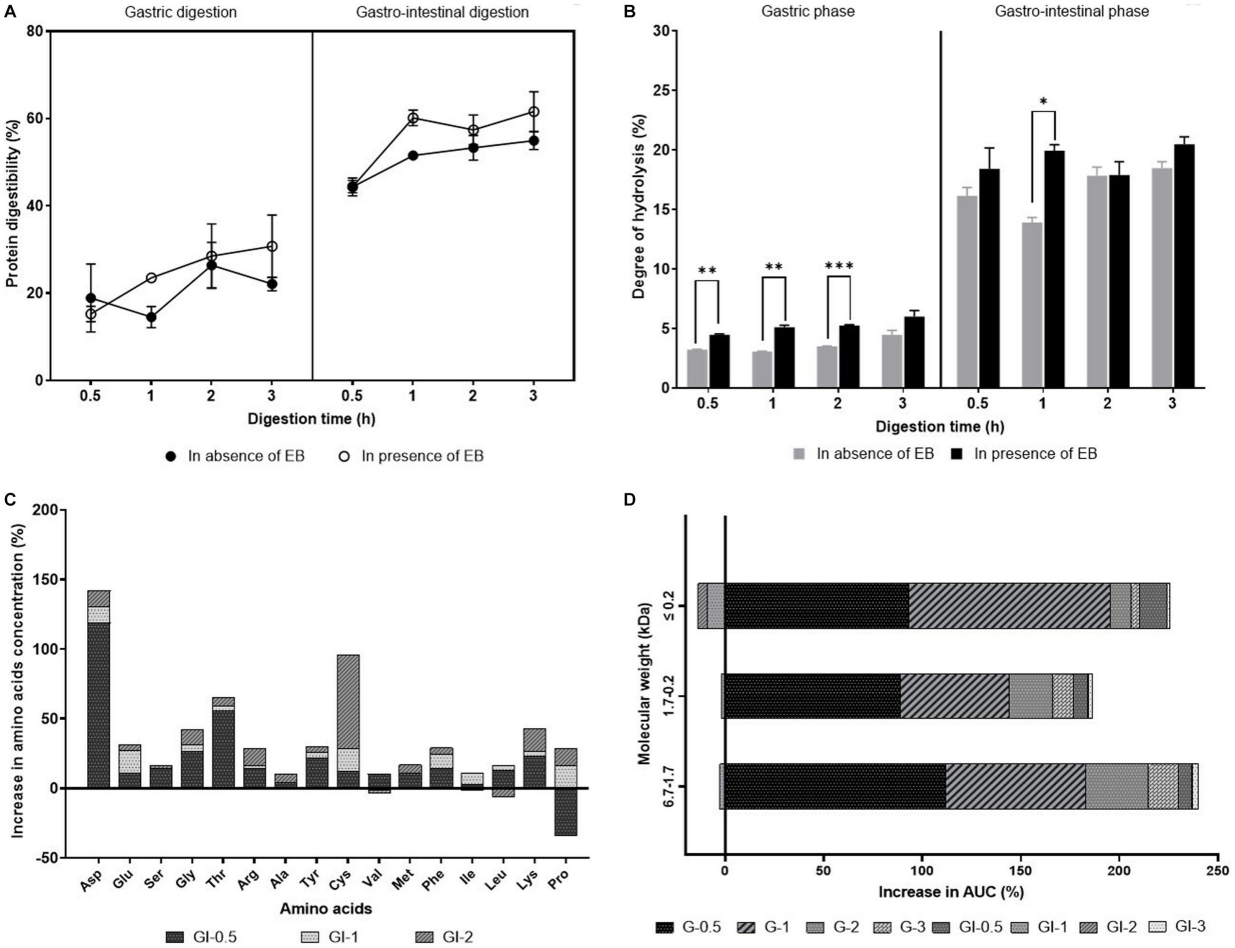
**(A)** Protein digestibility (%) and **(B)** Degree of hydrolysis (%) after gastric and gastro-intestinal digestion of the canine food in absence and presence of enzyme blend. **(C)** Increase in amino acid concentration (%) after gastro-intestinal (GI) digestion (digestion time—0.5, 1, and 2 h) of the canine food in presence of enzyme blend. **(D)** Increase in AUC (%) after gastric (G) and gastrointestinal (GI) digestion (digestion time—0.5, 1, 2, and 3 h) of the canine food in presence of enzyme blend. Values are represented as mean ± standard deviation. Two-way ANOVA with Tukey’s multiple-comparison test was used to determine the *p* value. ^*^, ^**^, and ^***^ represent significant difference at *p* ≤ 0.05*, p* ≤ 0.005, and *p* ≤ 0.001, respectively.

In this study, a simulated semi-dynamic *in vitro* digestion model was used to evaluate the effect of EB (DigeSEB super pet) on canine food digestibility. The protein digestibility value obtained by our model was 55 ± 2.1% (Figure 1A), similar to the value obtained by Smeets-Peeters et al. (3) using a dynamic model (fast transit time), i.e., 62%. The variation in digestibility can be attributed to the difference in food composition as well as the nature of the model (semi-dynamic vs. dynamic). Digestion is a complex process that involves physicochemical, mechanical, and microbial parameters, which play a paramount role in canine health. *In vivo* canine food digestibility has been reported previously (31, 32) in literature. However, the restrictions imposed on the *in vivo* studies in canines are stringent due to ethical, regulatory, societal, and economical pressures. Alternatively, *in vitro* digestion models such as static and dynamic canine digestion model have also been used previously in various studies, including on protein digestibility, calcium availability (3), organic matter and energy digestibility (33), selenium accessibility (34), *in vitro* dissolution of formulation (35), the effect of supplementation of larvae meal in canine food on digestibility (36), and the effect of thermal processing on the digestibility of raw chicken meat (37). Although dynamic models closely mimic the complex nature of the digestive system, its laboratory practicality is constricted. On the other hand, the semi-dynamic model described in this study is simple, inexpensive, time saving, reproducible, and feasible in any lab. Although the results are not directly comparable to *in vivo* data in dogs, it can be used as a screening step/predictive system to optimize/compare the formulation/product before proceeding to the clinical study in dogs. The results of such *in vitro* studies can also envisage a prospective clinical study.

A dynamic canine digestion model developed by Smeets-Peeters et al. (3) was computer controlled to simulate the pH, transit time, and secretion of digestive juices. While in this study, the semi-dynamic digestion model was manually altered for pH, addition of electrolytes, and enzymes based on the information (pH over the time, composition and concentration of electrolytes, and enzymes at any given time point) cumulated from the dynamic model. The pH was adjusted, and digestive juices (electrolytes and enzymes) were routinely added to the gastric phase post every 0.5 h. The intestinal phase, however, was static in nature—unlike the one used in the dynamic digestion model. While the dynamic digestion model spanned over 6 h consisting of various gastric and intestinal transit times, the semi-dynamic model differentiated into 3 h of gastric phase and 3 h of intestinal phase. Unlike dynamic digestion model, semi-dynamic digestion model does not include membrane absorption hence can not estimate/predict the bioavailability of the nutrients.

### 3.2. Effect of enzyme-blend supplementation in canine food on the dry matter and energy digestibility

**Figure 2:**
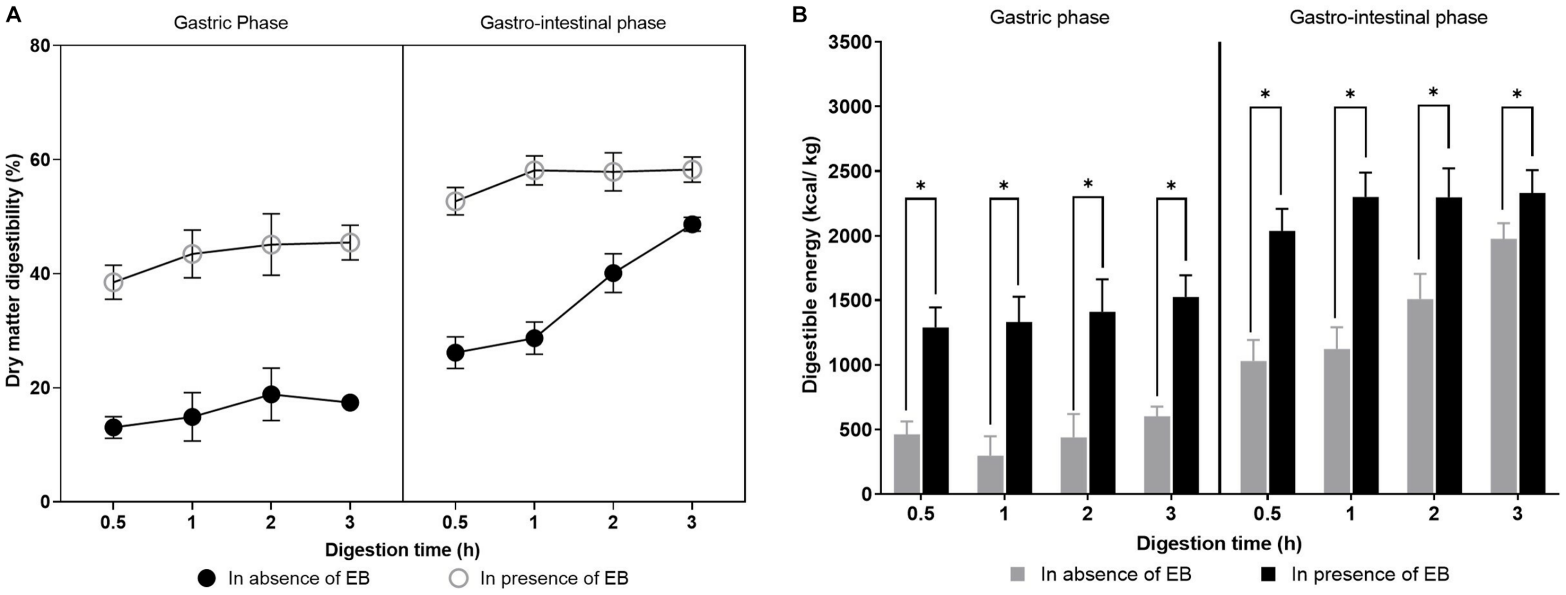
**(A)** Dry matter digestibility (%) and **(B)** Digestible energy (kcal/kg of sample) of the canine food in absence and presence of enzyme blend at gastric and gastro-intestinal phase. Values are represented as mean ± standard deviation. Student’s *t-test* was used to determine *p* value. ^*^represents a significant difference at *p* ≤ 0.05.

Dry matter digestibility and the digestible energy of the dry extruded canine food were determined in the absence and presence of EB supplement (DigeSEB Super Pet). In the absence of EB, dry matter and energy digestibility increased slowly during gastric digestion but rapidly during the gastrointestinal digestion. Alternatively, the supplementation of EB contributed to the increased digestibility during the gastric digestion itself, indicating the improved digestion of food. The EB could enhance the dry matter digestibility from 48 to 58% (*p <* 0.05) and energy digestibility from 1,975 to 2,331 kcal/kg (*p <* 0.05) post complete gastrointestinal digestion of the canine food (Figure 2). Enzyme blend supplements containing amylase, protease, and lipase assist the endogenous digestive enzymes in the breakdown of macromolecules to release the nutrients from the food matrix, which in turn aids in increasing food digestibility and availability of nutrients for absorption. In prior *in vivo* studies, exogenous enzyme supplementation had not shown any effect on the canine food digestibility (18). The enzyme performance is dependent on its activity and specificity; hence, the careful selection of enzymes is necessary for its effect on digestibility. This also highlights the importance of an *in vitro* simulated digestion model in the optimization of formulation/product prior to designing an *in vivo* study.

Bourreau et al. (38) has previously explained that a high gastric emptying rate might overload the small intestine of smaller canines due to discharge of inadequately pre-digested food particles. These food particles are generally less susceptible to intestinal enzymatic hydrolysis and remain undigested. The undigested food then serves as a nutrient source for microbiomes through colonic fermentation (saccharolysis, proteolysis, and lipolysis). The metabolites produced during such colonic fermentation can have beneficial/detrimental effects on the host, depending on their nature. Proteolytic putrefaction produces proinflammatory uremic toxins, which have a negative impact on the host (39). Increment in dry matter digestibility of canine food observed after supplementation of EB can reduce the flow of undigested material to the colon and can further reduce the potential harmful impact on the host.

### 3.3. Effect of enzyme blend supplementation in canine food on the protein digestibility

Protein is an indispensable macronutrient required for optimum growth and maintenance. After digestion, smaller peptides and amino acids are released from proteins, which get absorbed in the intestine where they serve as an energy source and provide components necessary for metabolic functions. However, if they remain undigested, protein reaches the colon and negatively influences the canine intestinal ecosystem. It increases the ammonia levels, reduces the volatile fatty acids, lowers the lactobacilli and enterococci, and increases the *Clostridium perfringens* (40). In the absence of EB, protein digestibility was 22.1 ± 1.6% (gastric phase) and 54.9 ± 2.1% (gastrointestinal phase), which increased to 30.7 ± 7.1% (gastric phase, *p >* 0.1) and 61.6 ± 4.5% (gastrointestinal phase, *p >* 0.1) in the presence of EB (Figure 1A). The degree of hydrolysis (DH) is the measure of protein hydrolysis during digestion. Higher DH correlates with more solubility and higher availability of the protein for the absorption. The digesta obtained in the absence of EB showed a DH of 4.4 ± 0.4% and 18.4 ± 0.6% in the gastric and gastrointestinal digestion, respectively, whereas in the presence of EB, it increased to 5.9 ± 0.5% (*p <* 0.1) and 20.4 ± 0.6% (*p <* 0.1), respectively (Figure 1B). The DH data at 2 h gastro-intestinal digestion was not in line with the trend over the digestion time, which might be due to an error while sampling the reaction mixture. External enzyme supplementation of protease has shown increased apparent ileal crude protein and amino acid digestibility of over processed soybean meals in boilers (41). The improved (though not statistically significant) protein digestibility and hydrolysis observed in our study was owing to the presence of proteases in the supplements that work complementary to the endogenous proteases.

The nutritive value of the protein is dependent on the bioavailable peptides and amino acids. The amino acid profile of the digesta revealed that the EB supplement could increase the indispensable amino acid release by 15.62% within 0.5 h GI digestion (Figure 1C). Moreover, it also increased the release of phenylalanine, tyrosine, cysteine, and methionine content by 14.5, 21.71, 12.1, and 10.6%, respectively. The molecular weight distribution of the digesta demonstrated that the EB supplement increased the release of lower molecular weight peptides in the gastric stage itself, corroborating with the higher protein digestibility in the gastric phase (Figure 1D). The smaller molecular weight peptides are easily absorbed in the intestine (28). Overall, the EB supplement was found to increase the protein digestibility, release of lower molecular weight peptides, and free amino acids in the canine food. Previously amino acid supplementation had shown to reduce hair loss (42), induce intense and darker hair coat colors (43), and promotes normal cardiac function (44) in canines.

### 3.4. Effect of enzyme blend supplementation in canine food on non-fibrous carbohydrate digestibility

Carbohydrates are a major part of canine food that provide energy and fibers (45). The composition and structure of carbohydrates affects their digestibility. High oil maize, broken rice, sorghum, and millet showed better digestibility and greater metabolizable energy for canines than wheat bran, maize germ, and rice bran (46). In the current study, the effect of the EB supplementation on the NFC digestion was studied in terms of reducing sugars and glucose released in the digesta. In absence of EB, the total reducing sugar was 9.8 ± 0.1 mg/g at 0.5 h of gastric digestion and reached 101.8 ± 0.1 mg/g at end of the digestion. While that in presence of EB, it was 151.5 ± 5 mg/g at 0.5 h of digestion and reached 210.1 ± 3.5 mg/g at the end of the digestion. The increased reducing sugar concentration indicated improved NFC digestion (*p* < 0.005; Table 1). Furthermore, the glucose release was 6.3 ± 0.3 and 30.5 ± 0.1 mg/g in the absence and presence of EB, respectively, at the end of the digestion (*p* < 0.0001; Table 1).

**Table 1:** Total reducing sugar release (mg/g of sample) and glucose release (mg/g of sample) in absence and presence of enzyme blend (EB).

Reaction time (h)	Total reducing sugar release (mg/g of sample)						Glucose release (mg/g of sample)		
	Gastric phase			Gastro-intestinal phase			Gastro-intestinal phase		
	Absence of EB	Presence of EB	*p* value	Absence of EB	Presence of EB	*p* value	Absence of EB	Presence of EB	*p* value
0.5	9.8 ± 0.1	151.5 ± 5^b^	0.0006	17.8 ± 1.1^d^	186.0 ± 9.8^b^	0.0017	4.8 ± 0.28	26.5 ± 1.5^a,b^	0.0024
1	9.1 ± 0.5	174.4 ± 1.9^a^	0.0001	38.2 ± 2.5^c^	188.0 ± 12^b^	0.0033	5.1 ± 0.22	23.9 ± 0.8^b^	0.001
2	9.0 ± 0.4	163.5 ± 5.6^a,b^	0.0006	72.3 ± 1.4^b^	188.1 ± 0.9^b^	0.0001	5.9 ± 0	27.3 ± 0.4^a,b^	0.0001
3	13.1 ± 0.2	170.4 ± 4.7^a^	0.0005	101.8 ± 0.1^a^	210.1 ± 3.5^a^	0.0005	6.3 ± 0.3	30.5 ± 0.1^a^	<0.0001

Moisture was not subtracted while calculating nutrient release. Data represented as mean ± standard deviation. Two-way ANOVA with Tukey’s multiple-comparison test was used to determine *p* value. Superscript lowercase letters present significant differences (*p* ≤ 0.05) between the samples of the same column, whereas the value of *p* represents a statistical relation between the samples in the same row.

Results demonstrated that EB supplementation promotes starch degradation as shown by the 2-fold increase in reducing sugar and 5-fold increase in glucose concentration. Amylase present in the EB supplement is hypothesized to work simultaneously or sequentially with pancreatic amylase in starch degradation to improve the digestion of NFC, which might have potential in management of the hypoglycemia in pets (47).

### 3.5. Effect of enzyme blend supplementation in canine food on the total antioxidant capacity

**Figure 3:**
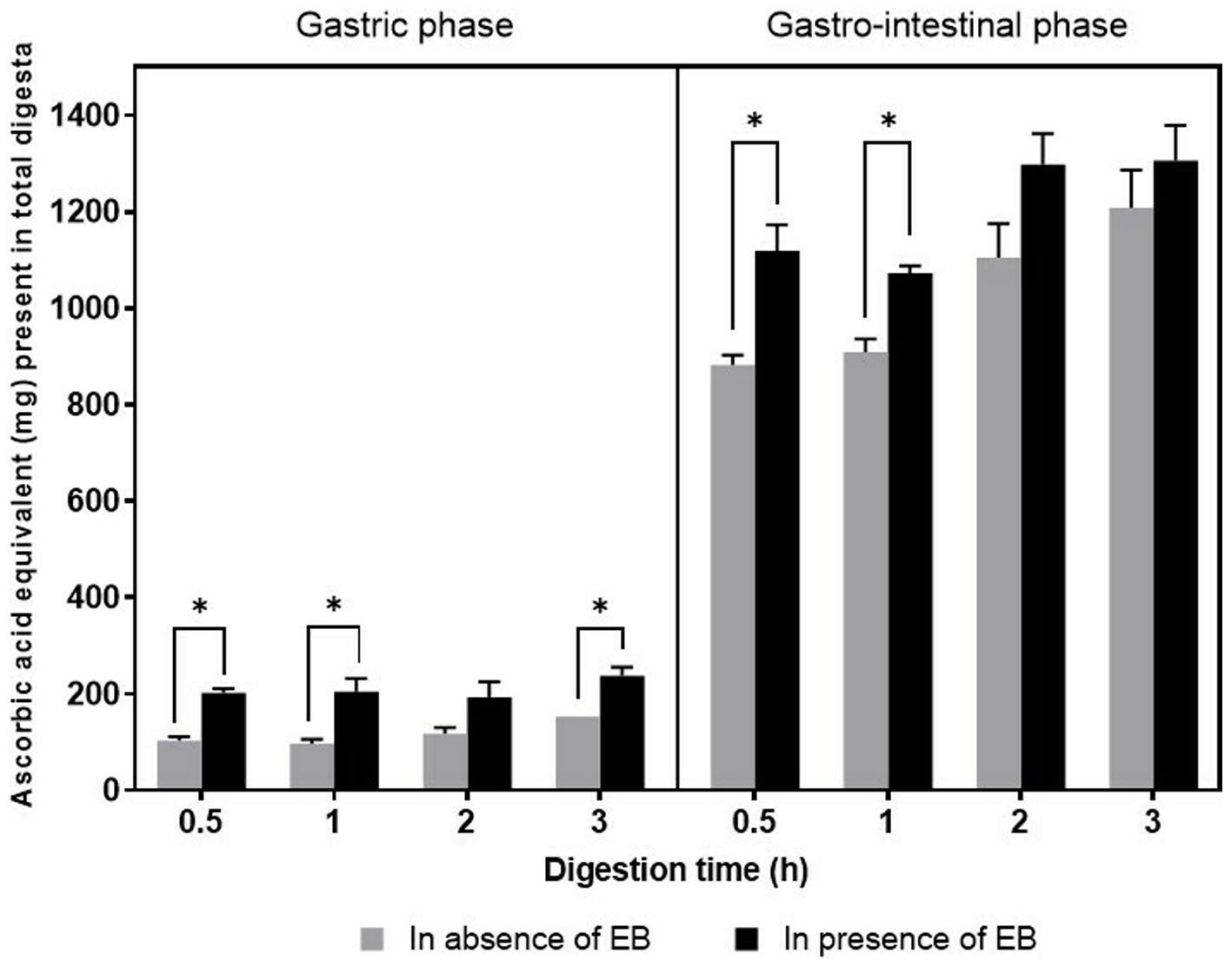
Total antioxidant capacity [ascorbic acid equivalent (mg) present in total digesta] after gastric (G) and gastro-intestinal (GI) digestion of the canine food in absence and presence of enzyme blend. Two-way ANOVA with Tukey’s multiple-comparison test was used to determine *p* value. ^*^represents a significant difference at *p* ≤ 0.05.

The total antioxidant capacity of the canine diet is useful to the regulation of the health status of the pet. The total antioxidant capacity of the digesta obtained in the absence and presences of EB was evaluated using a phosphomolybdate assay. The TAC of the digesta increased in the gastrointestinal phase compared to the gastric phase, which may be due to the larger degradation of the macromolecules in the gastrointestinal phase to release the antioxidants from the complex food matrix. The TAC of the digesta in the presence of EB was significantly higher than in the absence of EB at 0.5 h (*p* < 0.05) and 1 h (*p* < 0.05). Although not significantly different, at the end of the digestion (3 h), the TAC of the digesta in the presence of EB [1,306 ± 74 ascorbic acid equivalent (mg)] was higher than in the absence of the EB [1,208 ± 78 ascorbic acid equivalent (mg); Figure 3]. At the end of the digestion, EB supplement contributed to the increase in TAC by 8.1%. EB supplemented in the canine diet was found to release maximal antioxidant from the food matrix, potentially playing a vital role in the management of the oxidative status of the pet (48, 49). Antioxidant supplements in the diet of 62 Alaskan sled canines has previously shown resistance to exercise-induced oxidative damage (48). Antioxidant blends of vitamins, minerals, and carotenoids supplemented in the canine diet showed increased circulation of antioxidants and reduced DNA damage (49).

The positive impact of EB supplementation in the canine diet was illustrated in the simulated *in vitro* semi-dynamic digestion model with respect to dry matter and energy digestibility and release of macronutrients and antioxidants. Though *in vivo* studies in pets might shed more light on the importance of the enzyme supplements in the pet food, this model is beneficial as a predictive system in screening/optimizing formulations/products before proceeding for *in vivo* trials. However, this model has a few limitations, such as the results not being directly comparable with the *in vivo* results—it fails to incorporate the complexity of the dynamic nature of the digestive system, it cannot mimic the neuro-hormonal feedback mechanism, and it is unable to elucidate the bioavailability of the nutrients.

## 4. Conclusion

A simulated semi-dynamic *in vitro* canine digestion model was used to evaluate the effect of external enzyme blend supplementation in canine food on the digestibility. DigeSEB Super Pet; an enzyme blend supplementation, not only increased the dry matter and energy digestibility but also improved the protein and NFC digestion. Moreover, the total antioxidant capacity of the digested food was also found to be increased due to DigeSEB Super Pet. Overall, enzyme blend supplementation in the canine diet increased the food digestibility and the release of nutrients for absorption that would in turn ensure that the pet is adequately nourished.

## Data availability statement

The original contributions presented in the study are included in the article/Supplementary material, further inquiries can be directed to the corresponding author.

## Author contributions

SJ and AR: conceptualization, methodology, validation, and writing—review and editing. MJ and TG: formal analysis and data curation. SJ and TG: writing—original draft preparation and visualization. SJ: supervision. All authors contributed to the article and approved the submitted version.

## Conflict of interest

AR, SJ, MJ, and TG are paid employees of Advanced Enzyme Technologies, which has a corporate affiliation with Specialty Enzymes and Probiotics. Specialty Enzymes and Probiotics had no role in the study design and actual conduct of the study.

## Publisher’s note

All claims expressed in this article are solely those of the authors and do not necessarily represent those of their affiliated organizations, or those of the publisher, the editors and the reviewers. Any product that may be evaluated in this article, or claim that may be made by its manufacturer, is not guaranteed or endorsed by the publisher.
