# Supplementary material for: Modulation of digestibility of canine food using enzyme supplement: an in vitro simulated semi-dynamic digestion study
Source: Front Vet Sci. 2023 Aug 9;10:1220198. doi: 10.3389/fvets.2023.1220198 (PMC10445143; doi:10.3389/fvets.2023.1220198)
Supplement: Supplementary file 1 [file Data_Sheet_1.docx]

Supplementary Material

Modulation of digestibility of commercial canine food using enzymes supplement: *in-vitro* simulated semi-dynamic digestion study

Swati Jadhav^1*^, Tejal Gaonkar^1^, Mithila Joshi^1^, and Abhijit Rathi^1^

*** Correspondence:** Swati B. Jadhav: swati@advancedenzymes.com

# Supplementary Figures and Tables

## Supplementary Figures


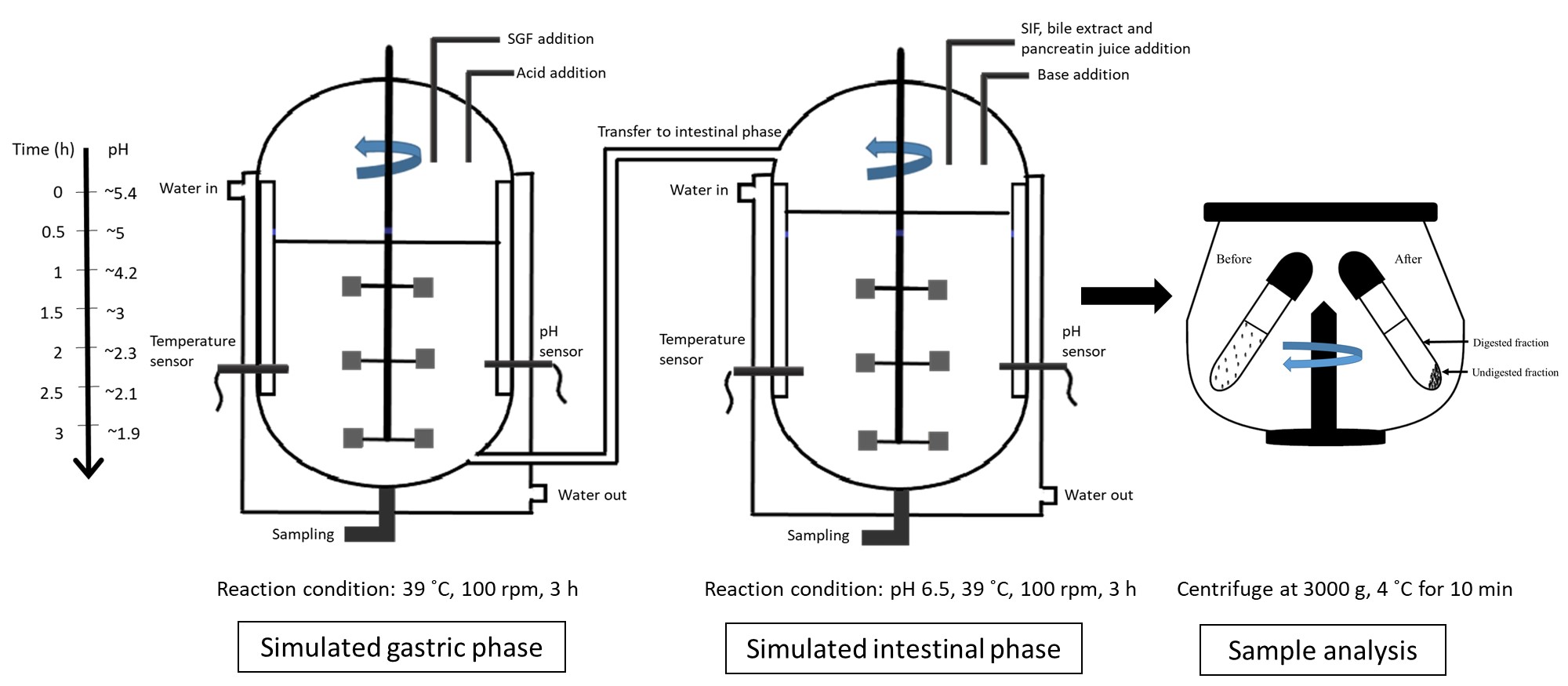


**Supplementary Figure 1.** Schematic representation of the semi dynamic *in-vitro* digestion model.

## Supplementary Tables

Supplementary Table 1: Composition of the digestive juices used in the semi-dynamic *in-vitro* digestion model

| **Digestive juice** | **Components** | **Concentration (g/L)** |
| --- | --- | --- |
| Simulated gastric fluid (SGF, pH 1.9) | NaCl | 3.5 |
|  | KCl | 1.3 |
|  | NaHCO_3_ | 0.25 |
|  | CaCl_2_.2H_2_O | 0.2 |
|  | Pepsin | 0.075 |
|  | Lipase | 0.09 |
| Simulated intestinal fluid (SIF) | NaCl | 7 |
|  | KCl | 0.5 |
|  | MgCl_2_.6H_2_O | 0.813 |
| Bile solution | Bile powder | 60 |
| Pancreatin solution | Pancreatin powder | 10 |
